# Associations of frailty with health care costs – results of the ESTHER cohort study

**DOI:** 10.1186/s12913-016-1360-3

**Published:** 2016-04-14

**Authors:** Jens-Oliver Bock, Hans-Helmut König, Hermann Brenner, Walter E. Haefeli, Renate Quinzler, Herbert Matschinger, Kai-Uwe Saum, Ben Schöttker, Dirk Heider

**Affiliations:** Department of Health Economics and Health Services Research, Hamburg Center for Health Economics, University Medical Center Hamburg-Eppendorf, Martinistrasse 52, Hamburg, 20246 Germany; Division of Clinical Epidemiology and Aging Research, German Cancer Research Center, Im Neuenheimer Feld 581, Heidelberg, 69120 Germany; Network Aging Research, University of Heidelberg, Bergheimer Straße 20, Heidelberg, 69115 Germany; Department of Clinical Pharmacology and Pharmacoepidemiology, University of Heidelberg, Im Neuenheimer Feld 410, Heidelberg, 69120 Germany; Institute of Social Medicine, Occupational Health and Public Health, University of Leipzig, Philipp-Rosenthal-Strasse 55, Leipzig, 04103 Germany

**Keywords:** Economic, Utilization of services, Frailty, Costs, Old age

## Abstract

**Background:**

The concept of frailty is rapidly gaining attention as an independent syndrome with high prevalence in older adults. Thereby, frailty is often related to certain adverse outcomes like mortality or disability. Another adverse outcome discussed is increased health care utilization. However, only few studies examined the impact of frailty on health care utilization and corresponding costs. The aim of this study was therefore to investigate comprehensively the relationship between frailty, health care utilization and costs.

**Methods:**

Cross sectional data from 2598 older participants (57–84 years) recruited in the Saarland, Germany, between 2008 and 2010 was used. Participants passed geriatric assessments that included Fried’s five frailty criteria: weakness, slowness, exhaustion, unintentional weight loss, and physical inactivity. Health care utilization was recorded in the sectors of inpatient treatment, outpatient treatment, pharmaceuticals, and nursing care.

**Results:**

Prevalence of frailty (≥3 symptoms) was 8.0 %. Mean total 3-month costs of frail participants were €3659 (4 or 5 symptoms) and €1616 (3 symptoms) as compared to €642 of nonfrail participants (no symptom). Controlling for comorbidity and general socio-demographic characteristics in multiple regression models, the difference in total costs between frail and non-frail participants still amounted to €1917; *p* < .05 (4 or 5 symptoms) and €680; *p* < .05 (3 symptoms). Among the 5 symptoms of frailty, weight loss and exhaustion were significantly associated with total costs after controlling for comorbidity.

**Conclusions:**

The study provides evidence that frailty is associated with increased health care costs. The analyses furthermore indicate that frailty is an important factor for health care costs independent from pure age and comorbidity. Costs were rather attributable to frailty (and comorbidity) than to age. This stresses that the overlapping concepts of multimorbidity and frailty are both necessary to explain health care use and corresponding costs among older adults.

**Electronic supplementary material:**

The online version of this article (doi:10.1186/s12913-016-1360-3) contains supplementary material, which is available to authorized users.

## Background

Life expectancy increases substantially in virtually all developed countries [1], leading to a larger number of older people living in these countries. Old age is accompanied by many geriatric phenomena that include, for example, multiple chronic conditions, also referred to as ‘multimorbidity’ [2, 3]. As the number of people in old age increases, many studies investigated the impact of multimorbidity on health care costs [4]. These studies find in general a positive association of multimorbidity and health care costs, stressing the importance of this phenomenon for the health care system.

Another medical phenomenon associated with age is people’s vulnerability to negative health outcomes and the general loss of resources. This phenomenon of frailty has increasingly received attention during the past decades. Thus, it has been shown that frailty is frequent in old age [5], and the number of frail people is expected to rise rapidly due to demographic change, stressing its importance for health care systems.

Despite the great attention in the gerontological field, there is no generally accepted definition of frailty [6–8]. Fried et al. proposed a definition of frailty that characterizes it as an independent phenotype differing from comorbidity and disability [9]. According to their definition, frailty is a clinical syndrome constituted by the co-occurrence of at least three of the following five criteria: unintentional weight loss, exhaustion, weakness, slow walking speed, and low physical activity. Fried et al. [9] as well as other studies [10] related frailty to certain ‘adverse outcomes’, for which the predictive validity of frailty has been investigated. For example, frailty has been found to be highly predictive for mortality [11–14]. Another adverse outcome potentially associated with frailty is increased health care utilization. Some studies examined the relationship between frailty and health care utilization, finding in particular an increased hospitalization rate among frail older adults [9, 15–21].

In order to extend these studies and provide evidence from a representative large population-based sample, we aimed at examining comprehensively the effect of frailty on health care utilization and corresponding costs in all important health care sectors, including inpatient services, outpatient services, pharmaceuticals, and nursing care. Thus, it was our goal to present cost estimates for frailty. In particular, the aims of this study were i) to investigate the relationship between frailty and health care costs in a large sample of older adults and ii) to determine the respective associations of the different frailty criteria and health care costs.

## Methods

### Sample

**Fig. 1:**
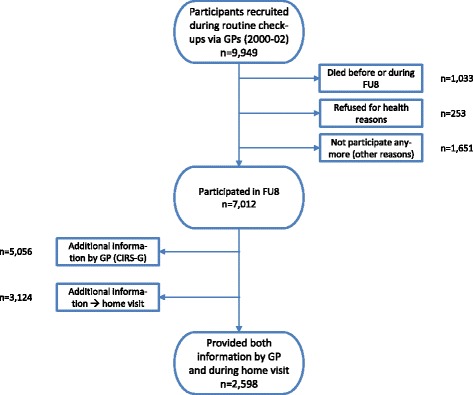
Flow chart of sample selection in follow-up 8 of the ESTHER study

The cross-sectional analyses presented in this manuscript are based on the 8-year follow-up wave of the “Epidemiological investigations on chances of preventing, recognizing early and optimally treating chronic diseases in an elderly population”, the ESTHER-Study. ESTHER is a large prospective observational cohort study of older Germans. For this study, 9949 patients, aged 50–75 years, were recruited via their GPs in the Saarland, Germany between July 2000 and December 2002. Participants’ socio-demographic and lifestyle factors were collected by standardized questionnaires and clinical data by their general practitioners (GPs) and study physicians. Follow-up questionnaires were sent to the participants and their GPs 2, 5 and 8 years after recruitment. From baseline-recruitment to the 8-year follow-up, 1033 participants had died, 1904 had discontinued further active participation, thereof 253 for health reasons. The questionnaire of the 8-year follow-up was provided either by the GPs or by the patient for 7012 study participants. This corresponds to an 80.9 % response rate among survivors still mentally and physically able to respond. Information from both the GP’s and the patient’s questionnaires was collected for 5057 of the participants. At the 8-year follow-up, participants were invited in written form by the study secretariat to take part in detailed geriatric assessments that were conducted at the participants’ homes by trained study doctors. Participation was optional and reasons for denying participation were not collected. For the geriatric assessments several data were collected, e.g. on (instrumental) activities of daily living, body weight, height, cognitive abilities, measures and constitution. Moreover, the assessments included interviews to collect the health service use as well as measurement of frailty symptoms to be used for the analysis in the present study. 3124 study participants in whom home visits were completed accepted the additional offer. Further detailed information about ESTHER can be found elsewhere [14, 22–25]. All following analyses were based on the subsample of the 8-year follow-up with comprehensive geriatric assessment at the home visits and for whom data provided by the GP were available (*n* = 2598 participants). Figure 1 shows the sample selection process.

### Ethics, consent and permission

The ESTHER study has been approved by the ethics committees of the Medical Faculty of the University of Heidelberg and the Medical Association of Saarland. A signed statement of informed consent has been obtained from all participants included in the ESTHER study.

### Health care costs

As recommended [26], we adopted a societal perspective for calculating health care costs. The calculation of health care costs generally contains three steps [27]: 1) Identifying relevant cost components; 2) collecting resource consumption for these identified components; 3) valuing the resource consumption. For the first step, we considered the following health care sectors as highly relevant for the cost calculation: inpatient treatment, outpatient treatment including physician and non-physician outpatient treatment, medical supplies, dental prostheses, pharmaceuticals, and nursing care, the latter including both formal and informal care.

For the second step, data on the resource consumption was collected retrospectively for a 3-month period using a previously developed and tested questionnaire [28, 29]. The questionnaire comprehensively listed health care goods and services that older adults could potentially use in order to minimize recall bias; it is available as Additional file 1. Cognitive abilities of participants were on average very good as indicated by a mean MMSE [30] of 28.2 points (SD: 2.1). Only 5.3 % of participants scored less than 25 points and can thus be classified as at least mildly cognitively impaired. This suggests that the recall period of 3 month was appropriate at least for the vast majority of participants. The questionnaire was handed over to and filled out by the participant during the geriatric assessments at his or her home. For the inpatient sector, the questionnaire recorded the number of days in hospital. Outpatient medical services were measured in terms of physician visits; medical supplies, such as walkers, hearing aids etc., in terms of their respective quantities. Pharmaceuticals were directly recorded by means of barcode readers by the study physicians. Nursing care included professional home care in terms of hours, and nursing home care in terms of days in institutionalized homes. The use of informal care was recorded by asking participants, i.e. the potential receivers of informal care, whether they utilized aid for reasons of high age or bad health provided by spouses, other family members, friends or neighbors. The analyses did not consider indirect costs. A majority of the participants had already reached the retirement age, for the remaining ones, we focused on direct health care costs.

The third step contained the monetary valuation of the quantities of the resource used with corresponding unit costs for inpatient care [31–33], outpatient care [34–38], medical devices [34, 39, 40], pharmaceuticals [41], nursing home care [42], professional home care and informal care [43, 44]. The costing process is depicted in Table 1. Further details concerning the costing for the 8-year follow-up-wave of ESTHER have also been reported in detail elsewhere [45]. Costs were calculated based on prices of the year 2009.

**Table 1:** Recorded resource utilization and source of valuation (Adapted from [45])

Health care sector	Example of resources	Unit	Soource
In-patient treatment	Stays in general hospitals, specialized psychiatric and neurological hospitals or rehabilitation clinics (including day patient treatment)	Days in hospital	Calculated costs of care per day by type (Federal Statistical Office, German Hospital Federation, Statutory Pension Insurance Fund) [31–33]
Out-patient physician treatment	Treatment by GPs, specialists and out-patient clinics	Number of visits	Calculated costs per contact, by specialization [34]
Other out-patient treatment	E.g. physiotherapy, massage, occupational therapy, non-medical practitioner	Number of visits	Reimbursement schedules (Statutory health insurance funds; [35–37], calculated costs per contact [34], by type, schedule of fees (Federal office of administration) [38]
Medical supplies and dental prostheses	E.g. walkers, incontinence pads, hearing aids, surgical stockings, dental bridge, crown	Quantity	Reimbursement schedules (Statutory health insurance funds, Federal Association of Panel Dentists; [39, 40], calculated costs per item [34], by type
Pharmaceuticals	Specific products (including trade name, drug code, package size, pharmaceutical form, dosage)	Quantity	Pharmacy retail prices (MMI-Pharmindex, Medizinverlag Medizinische Medien Informations GmbH (MMI, Neu-Isenburg) [41]
Nursing home care	Nursing home stays (residential and day care)	Days	Calculated costs of care per day (Federal Statistical Office) [42], by type
Professional home care	Care and assistance provided by professional nursing services and other paid help, differentiated by type (e.g. basic care, assistance with cleaning, shopping, financial matters etc.) and limited to care or assistance required owing to illness or age	Hours	Hourly gross wage rate plus non-wage labor costs for employees in the domain of care and assistance for the elderly or handicapped (Federal Statistical Office) [43, 44]
Informal care	Care and assistance provided by family or friends, differentiated by type and limited to care or assistance required owing to illness or age	Hours	Replacement cost method: Hourly gross wage rate plus non-wage labor costs for employees in the domain of care and assistance for the elderly or handicapped (Federal Statistical Office) [43, 44]

### Frailty

We create a frailty index according to Fried et al. [9] based on the five criteria: weakness, slowness, exhaustion, weight loss, and physical inactivity. The index was created by assigning a score of one if one of the five criteria was met, two if two of the five were met, etc. Persons with one or two criteria fulfilled were referred to as ‘pre-frail’, those with at least three as ‘frail’. Trained study physicians measured the symptoms at participants’ homes. We operationalized the five criteria as described elsewhere in detail [23]. Weakness was measured as grip strength, assessed three times by use of a Jamar hand dynamometer. For the analyses, the best result of the three measurements was used. Slowness was measured using the Short Physical Performance Battery (SPPB) [46]. The average walking distance per second in m/s was derived from the time to walk 3 m. Exhaustion was measured based on two questions [9] of the Center for Epidemiologic Studies Depression Scale (CES-D) [47]: “I felt that everything I did was an effort” and “I could not get going”. Weight loss was operationalized as an unintentional loss of at least 5 kg during the last year. Physical activities were assessed by means of the Physical Activity Questionnaire for the Elderly (PAQE) [48]. In contrast to Fried et al., we used population-independent cut-off points for the individual frailty criteria [23] in order to provide results that are comparable to other samples.

### Comorbidity

In order to calculate the net impact of frailty on health care costs, i.e. the impact irrespective of the potential influence of chronic conditions, cost estimates were adjusted for the influence of comorbidity. As measure of comorbidity, we considered the Cumulative Illness Rating Scale for Geriatrics (CIRS-G) [49, 50]. The CIRS-G is a generic measure of comorbidity consisting of 13 somatic categories and one psychiatric category. Each category is weighted according to severity by the GP with 0 points (‘no problem’) to 4 points (‘very severe problems’). The sum score over each category’s points defines the CIRS-G that can range from 0 to 56.

### Other variables

The statistical models include the covariates age, sex, marital status, educational level and type of health insurance. Marital status distinguished between single, married, divorced, and widowed. The educational level was operationalized as duration of primary and secondary school education, consisting of three classes: ≤9 years, 10–11 years, and ≥12 years. Participants’ type of health insurance could vary between statutory health insurance and private health insurance, reflecting the dualistic system of health insurance in Germany. While the majority of about 90 % of the overall German population are a members of a Statutory Health Insurance (SHI), the remaining 10 % are privately insured. Mandatory members of a SHI are especially employees under an income-threshold and, in general, their spouses and children. Both types of health insurance offer comprehensive coverage of costs in the inpatient and outpatient physician setting, and of prescription drugs. Moreover, permanent nursing care (both ambulatory and in nursing homes) is partially reimbursed by compulsory long-term care insurances.

#### Statistical analyses

All statistical analyses were done using Stata Release 14. The relationship between frailty as well as the single frailty symptoms and health care costs was analyzed using generalized linear models (glm) with log link function and gamma distribution, a commonly used type of model for skewed cost data [51]. For the health care sectors of inpatient treatment and nursing care, there was a vast majority of participants not using any services in these sectors during the 3 months preceding the interviews. This results in distributions of sectoral costs with many zero values, while the cost data of the users were positively skewed. Therefore, we calculated two-part models [52, 53], with the first part specified as ‘logit’, and the second part as glm with log link function and gamma distribution. For both total and sectoral regression models, we present the marginal effects at means of all variables obtained from the ‘twopm’ command [54], which can be interpreted in the same metric as the dependent variable, i.e. 3-month health care costs.

In order to examine robustness of regression models, the possible effect of frailty on sectoral and total health care costs was also investigated using multiple Tobit regression models. The characteristics of our dependent variables, in particular inpatient and nursing care cost with many zero values, were similar to those of censored data, for which Tobit models are recommended [55].

All regression analyses were performed with and without including the comorbidity status, operationalized as the CIRS-G. Thus, regression analyses firstly show the results of excess costs associated with frailty irrespective of morbidity status. Then, adjusting for morbidity status, they show the net excess costs associated with frailty with constant comorbidity. These models were run separately since frailty and comorbidity are overlapping concepts [19] with unclear causal interferences. The correlation between the frailty index and CIRS-G was 0.3.

We performed complete case analyses (listwise deletion) because missing values in any variable occurred in only 90 out of 2598 observations (3.5 %). The level of significance was set to α = 0.05.

## Results

### Study sample

Table 2 shows the sample characteristics for 2598 participants of the 8-year follow-up of the ESTHER study aged on average about 70 years. 52 % of participants were female, 73 % married, 17 % widowed, others divorced or single. The majority of participants had a primary and secondary school education of not more than 9 years. Ninety-two percent were insured by a statutory health insurance.

**Table 2:** Sample characteristics (*N* = 2598)

Characteristic		N	%
Age in years		Mean: 69.55	SD: 6.23
Sex	- female	1339	51.5 %
	- male	1259	48.5 %
Marital status	- single	84	3.3 %
	- married	1874	72.8 %
	- divorced	177	6.9 %
	- widowed	4,8	17.0 %
Education	≤9 years	1708	66.7 %
	10–11 years	451	17.6 %
	≥12 years	402	15.7 %
Type of health insurance	- SHI	2375	92.1 %
	- PHI	203	7.9 %
Frailty	- Non-frail (0)	876	33.8 %
	- Pre-frail (1)	1034	39.9 %
	- Pre-frail (2)	472	18.2 %
	- Frail (3)	142	5.5 %
	- Frail (4)	61	2.4 %
	- Frail (5)	4	0.2 %
Low physical activity	no	2078	80.0 %
	yes	520	20.0 %
Exhaustion	no	2290	88.1 %
	yes	308	11.9 %
Weight loss	no	2479	95.8 %
	yes	110	4.2 %
Slowness	no	1665	64.1 %
	yes	933	35.9 %
Weakness	no	1788	68.8 %
	yes	810	31.2 %

*SD* Standard deviation, *PHI* Private Health Insurance, *SHI* Statutory Health Insurance

The frailty syndrome with population-independent cut-points could be calculated for 2589 individuals, as the weight loss could not be documented for 9 observations. For one individual, both the status of potential weight loss and the symptom of physical activity were missing. The remaining symptoms of exhaustion, slowness and weakness were documented completely for all participants. About one third of the sample was non-frail. A majority of 58 % of participants had a score of ‘1’ or ‘2’ on the frailty index and was therefore classified as pre-frail. The frailty syndrome was present in 8 % of participants who scored at least 3 points. The prevalence of slowness was highest among frailty symptoms, with about 36 % being affected, followed by weakness (31 %) and low physical activity (20 %). In contrast, exhaustion occurred in only 12 % and weight loss in only 4 % of participants.

### Descriptive analyses

Table 3 shows the mean health care costs per respondent for a 3-month period by health care sector and in total. Mean total costs were €874 (SD: €2197), thereof 44 % inpatient costs, 31 % outpatient costs, and 19 % costs of pharmaceuticals. Nursing care contributed only 5 % to total costs. An increasing frailty index score was associated with increased costs in all health care sectors as well as total costs. In particular, costs of inpatient care increased substantially with the frailty index score, amounting to €2104 in frail participants with an index ≥4 compared to €268 in non-frail participants. Non-frail participants utilized almost no nursing care services, resulting in mean costs of only €2 in this sector. In contrast, mean costs of nursing care for frail participants amounted to €262 (frailty index 3) or €672 (frailty index ≥4) in the considered 3 month period. Mean costs of pharmaceuticals steadily increased with the frailty index score, ranging from €128 (non-frail) to €400 (frailty index 4 or 5).

**Table 3:** Mean 3-month health care costs in € (2009 Values) by frailty status

Frailty-Index	N	Total (SD)	Outpatient (SD)	Inpatient (SD)	Nursing care (SD)	Pharmaceuticals (SD)
Non-frail (0)	876	642 (1546)	243 (500)	268 (1362)	2 (35)	128 (218)
Pre-frail (1)	1034	733 (1628)	281 (567)	286 (1396)	11 (196)	154 (278)
Pre-frail (2)	472	1014 (2322)	270 (380)	492 (2149)	30 (342)	221 (286)
Frail (3)	142	1616 (4002)	345 (718)	747 (3082)	262 (1237)	263 (246)
Frail (4&5)	65	3659 (5918)	483 (777)	2104 (5268)	672 (1894)	400 (679)
Low physical activity						
- yes	520	1283 (2919)	291 (526)	575 (2524)	151 (874)	266 (397)
- no	2078	771 (1963)	270 (534)	341 (1676)	15 (288)	145 (238)
Exhaustion						
- yes	308	1853 (4189)	367 (503)	990 (3520)	232 (1160)	265 (406)
- no	2290	742 (1725)	263 (263)	307 (1512)	16 (256)	156 (258)
Weight loss						
- yes	110	2435 (4254)	377 (639)	1762 (3969)	39 (257)	256 (574)
- no	2479	805 (2036)	270 (527)	328 (1708)	42 (479)	166 (261)
Slowness						
- yes	933	1099 (2815)	282 (488)	509 (2390)	103 (761)	206 (293)
- no	1665	747 (1746)	271 (556)	320 (1515)	7 (137)	149 (273)
Weakness						
- yes	810	1063 (2834)	315 (671)	474 (2320)	93 (755)	180 (283)
- no	1788	788 (1831)	257 (456)	349 (1639)	18 (250)	164 (279)
All	2598	874 (2197)	275 (532)	388 (1878)	42 (471)	169 (282)

*SD* Standard deviation

Regarding the five constituting symptoms of frailty, mean costs always increased if one of the symptoms was present. This applied to costs in all considered health care sectors and to total costs. The increase in costs was most pronounced for unintentional weight loss where mean total costs of affected participants were €1630 higher compared to participants without this symptom. This difference was mainly due to inpatient costs which differed by about €1400.

### Inferential analyses of frailty index

Table 4 shows the results of the multivariate regression models for total and sectoral 3-month costs per respondent as dependent variable, with frailty as main independent variable, presenting the marginal effects at means of all variables. All regressions models were estimated (1) without and (2) with controlling for comorbidity.

**Table 4:** Multiple regression analyses^a^ with health care costs as dependent variable and frailty status as main predictor variable

	Models without comorbidity (1)					Models with comorbidity (2)				
	Total	Inpatient^b^	Outpatient	Pharmaceuticals	Nursing care^b^	Total	Inpatient^b^	Outpatient	Pharmaceuticals	Nursing care^b^
Prefrail (1)	77.51	2.94	35.15	26.76	12.74	56.19	−10.18	33.15	17.57	10.14
	(73.88)	(63.68)	(23.38)	(10.95)*	(10.35)	(73.21)	(63.21)	(23.25)	(9.72)	(9.06)
Prefrail (2)	366.93	245.08	25.23	95.81	8.31	227.08	169.58	9.93	62.03	5.40
	(124.28)**	(125.43)	(29.65)	(19.11)***	(5.86)	(111.61)*	(112.66)	(28.71)	(15.34)***	(4.81)
Frail (3)	968.14	515.10	100.01	143.38	76.96	680.42	368.85	55.84	87.31	48.97
	(330.26)**	(261.58)*	(61.57)	(40.15)***	(47.75)	(273.12)*	(219.89)	(54.52)	(29.41)**	(33.38)
Frail (4 & 5)	3093.56	1946.88	234.64	303.25	334.93	1917.03	1278.81	161.10	123.96	181.00
	(1119.22)**	(847.00)*	(118.79)*	(95.03)**	(167.83)*	(772.18)*	(623.41)*	(101.48)	(50.99)*	(109.49)
Age	4.41	−0.50	1.07	1.20	1.02	1.12	−2.48	0.63	0.97	0.82
	(6.41)	(5.74)	(1.77)	(0.94)	(0.50)*	(6.07)	(5.43)	(1.73)	(0.81)	(0.43)
Sex	97.37	94.22	−22.32	24.34	3.08	25.51	56.32	−33.31	14.75	0.81
	(80.06)	(70.36)	(22.68)	(11.70)*	(5.17)	(74.73)	(66.00)	(22.17)	(9.83)	(4.36)
Single	−156.63	−42.12	−21.16	−21.51	1.01	−214.93	−77.50	−40.70	−40.24	−2.39
	(169.62)	(140.52)	(56.23)	(27.99)	(12.76)	(145.24)	(117.21)	(50.60)	(20.24)*	(8.72)
Divorced	120.59	102.07	−24.30	−7.93	−12.42	139.17	100.90	−22.10	−0.56	−10.79
	(172.23)	(145.20)	(39.22)	(21.62)	(5.12)*	(166.91)	(138.69)	(38.66)	(19.11)	(4.79)*
Widowed	28.50	66.15	6.63	−18.00	−4.55	13.45	53.71	−0.52	−18.47	−4.71
	(109.65)	(98.89)	(30.88)	(14.49)	(5.06)	(101.63)	(91.39)	(29.59)	(12.15)	(4.36)
Education:	7.73	−46.47	56.69	3.99	5.39	39.37	−33.93	62.43	9.68	4.94
- middle	(103.11)	(87.39)	(32.01)	(15.23)	(8.33)	(99.17)	(83.14)	(31.46)*	(13.13)	(7.37)
- high	−98.13	−73.63	−12.96	−26.15	−1.37	−73.33	−55.10	−12.03	−24.37	−0.47
	(101.48)	(92.30)	(29.50)	(14.25)	(5.66)	(96.79)	(89.59)	(28.53)	(11.88)*	(5.09)
Private HI	312.74	213.40	46.41	64.25	−7.67	348.30	219.27	54.34	78.70	−5.79
	(191.36)	(175.75)	(47.08)	(27.70)*	(4.25)	(188.13)	(170.07)	(46.77)	(25.17)**	(4.10)
Comorbidity	./.	./.	./.	./.	./.	46.69	19.52	8.07	10.45	0.71
(CIRS-G)	./.	./.	./.	./.	./.	(7.79)***	(5.61)***	(2.06)***	(1.00)***	(0.32)*
N	2508	2508	2508	2508	2508	2508	2508	2508	2508	2508

*CIRS-G* Cumulative Illness Rating Scale for Geriatrics

* *p* < .05 **p < .01 *** *p* < .001

^a^Marginal effects of all variables at their means are reported; predictions obtained from generalized linear models with log link and gamma distribution or from two part models when indicated. ^b^predictions obtained from two-part models with logit regression for the first part and log-gamma model for the second part

In the models without controlling for comorbidity (1), a frailty index ≥4 was statistically significantly associated with total costs (+€3094; *p* < .001) as well as with all sectoral health care costs. None of the other covariates was significantly associated with total costs. In particular, age was not associated with higher total costs when controlling for frailty, whereas age was strongly associated with total costs when the variable ‘frailty’ was removed from the regression model (model not shown here). Besides frailty, age was significantly associated with higher costs for nursing care, while being divorced was significantly associated with lower costs for nursing care.

In the models controlling for comorbidity (2), frailty was still associated with higher total costs. However, corresponding marginal effects were generally lower compared to the model without comorbidity. In the outpatient and the nursing care sector, there was no significant association between frailty and costs. In contrast, frailty index scores of 2, 3 or ≥4 were significantly associated with increased costs for pharmaceuticals. A frailty index ≥4 increased inpatient costs by about €1279. In all health care sector as well as in total, comorbidity was associated with increased costs.

### Inferential analyses of frailty symptoms

Table 5 shows the results of multiple regression analyses, but unlike Table 4, with the individual symptoms of frailty as the main independent variables instead of the frailty status.

**Table 5:** Multiple regression analyses^a^ with health care costs as dependent variable and symptoms of frailty as main predictor variable

	Models without comorbidity (1)					Models with comorbidity (2)				
	Total	Inpatient^b^	Outpatient	Pharmaceuticals	Nursing care^b^	Total	Inpatient^b^	Outpatient	Pharmaceuticals	Nursing care^b^
Low activity	302.39	137.64	2.75	98.58	36.96	98.51	40.34	−19.28	65.29	28.00
	(116.48)**	(98.30)	(27.06)	(18.90)***	(17.99)*	(96.52)	(81.47)	(25.26)	(14.85)***	(15.07)
Exhaustion	816.48	457.81	83.66	73.89	42.65	664.33	391.67	66.72	44.05	30.23
	(211.81)***	(165.88)**	(40.50)*	(22.85)**	(27.19)	(182.61)***	(148.53)**	(37.68)	(17.55)*	(21.54)
Slowness	93.23	69.43	−2.88	39.67	12.61	53.73	52.22	−10.03	22.42	8.73
	(83.15)	(72.80)	(22.44)	(12.16)**	(8.83)	(76.38)	(66.78)	(21.50)	(10.20)*	(7.89)
Weakness	58.60	−11.61	50.72	1.20	0.12	23.83	−32.06	44.39	−1.22	0.23
	(82.82)	(66.30)	(24.70)*	(11.76)	(4.09)	(75.67)	(60.47)	(23.60)	(10.01)	(3.97)
Weight loss	1329.28	1331.25	97.01	62.17	0.73	1131.58	1150.01	86.13	27.72	−0.92
	(471.93)**	(432.21)**	(68.77)	(36.62)	(7.08)	(407.97)**	(379.96)**	(64.65)	(26.77)	(5.13)
Age	4.28	−0.2	1.23	1.64	0.48	1.75	−2.02	0.82	1.22	0.40
	(6.25)	(5.53)	(1.76)	(0.92)	(0.34)	(5.84)	(5.17)	(1.71)	(0.80)	(0.30)
Sex	82.40	77.15	−17.43	21.64	3.31	0.36	37.21	−30.00	13.93	1.64
	(78.23)	(67.21)	(22.73)	(11.37)	(4.43)	(72.23)	(62.45)	(22.06)	(9.76)	(3.83)
Single	−132.22	−64.76	−10.93	−17.39	−0.55	−193.27	−93.65	−33.02	−37.61	−2.46
	(169.48)	(122.03)	(57.84)	(27.5)	(8.72)	(142.33)	(100.29)	(51.44)	(20.22)	(6.05)
Divorced	104.08	103.67	−28.22	−5.42	−7.04	117.11	107.55	−25.62	0.67	−6.37
	(162.86)	(138.62)	(38.3)	(21.08)	(3.99)	(154.80)	(132.55)	(37.55)	(18.91)	(3.86)
Widowed	26.50	53.21	7.63	−15.41	−3.82	4.50	38.67	−0.91	−17.14	−3.83
	(105.49)	(92.05)	(30.69)	(14.11)	(3.23)	(95.45)	(83.17)	(29.11)	(12.03)	(2.94)
Education:	7.20	−20.47	55.23	5.82	−0.13	34.29	−4.07	60.28	9.93	0.38
- middle	(99.37)	(84.79)	(31.70)	(14.75)	(4.49)	(93.83)	(80.71)	(30.86)	(12.94)	(4.33)
- high	−90.79	−39.58	−16.81	−20.38	3.93	−62.48	−23.18	−16.03	−21.01	4.06
	(99.65)	(91.88)	(29.16)	(14.11)	(7.46)	(94.06)	(87.48)	(27.98)	(11.97)	(7.02)
Private HI	373.25	237.29	45.97	65.01	−4.22	393.33	242.59	54.18	77.74	−3.12
	(195.27)	(177.47)	(46.66)	(26.87)*	(3.83)	(187.09)*	(170.37)	(46.03)	(24.72)**	(3.94)
Comorb.	./.	./.	./.	./.	./.	47.34	19.45	8.31	10	0.51
(CIRS-G)	./.	./.	./.	./.	./.	(7.49)***	(5.41)***	(2.03)***	(0.98)***	(0.28)
N	2508	2508	2508	2508	2508	2508	2508	2508	2508	2508

*CIRS-G* Cumulative Illness Rating Scale for Geriatrics

* *p* < .05 **p < .01 *** *p* < .001

^a^Marginal effects of all variables at their means are reported predictions obtained from generalized linear models with log link and gamma distribution or from two part models when indicated. ^b^predictions obtained from two-part models with logit regression for the first part and log-gamma model for the second part

Without controlling for comorbidity (1), there was a statistically significant association between each single symptom of frailty and total costs except for ‘slowness’ and ‘weakness’. Among the three symptoms, unintentional weight loss was most strongly associated with total costs with higher total costs of €1329 (*p* < .01) compared to no weight loss. The symptom of exhaustion had the second highest on total costs (+€816; *p* < .001) followed by low physical activity (+€302; *p* < 0.01). With respect to inpatient costs, besides the effect of unintentional weight loss, only exhaustion had a statistically significant positive effect. In the outpatient sector, exhaustion and weakness were statistically significantly associated with costs. For nursing care only ‘low activity’ was statistically significantly associated with costs.

When controlling for comorbidity (2), the marginal effects of frailty symptoms generally decreased as compared to the previous models. Yet ‘exhaustion’ and ‘weight loss’ were still significantly associated with total costs and inpatient costs. The associations of ‘low activity’, ‘exhaustion’ and ‘slowness’ with pharmaceutical costs found in models (1) persisted in models (2) with their estimated marginal effect being somewhat decreased.

### Sensitivity analyses

We ran Tobit regression models analogous to the glms in Table 4 and 5 in order to test for the robustness of the results. The Tobit models, in general, confirmed the results of the glms. All marginal effects related to frailty that reached the level of significance in the glms did so in the Tobit models. In the Tobit model controlling for comorbidity, frailty index scores of 3 and ≥4 were significantly associated with total costs, with marginal effects of €784 and €2601, respectively. Of the single symptoms of frailty, exhaustion and weight loss were positively associated with total costs when controlling for comorbidity in the Tobit models. In addition, the association of low activity and total costs that was shown in the glm only without controlling for comorbidity, remained in Tobit model after controlling for comorbidity in the (*p* < .01).

## Discussion

This study analyzed the relationship between frailty and health care costs in a cross-sectional design of a population-based sample of community-dwelling older Germans. Frailty was operationalized by the five criteria suggested by Fried et al. (weakness, slowness, exhaustion, unintentional weight loss, and physical inactivity), using population-independent cut-off-points.

Frailty was strongly associated with total health care costs with this association persisting in regression models that additionally controlled for comorbidity. In particular, the frailty index was associated with inpatient costs and pharmaceutical costs, with the latter association also persisting after controlling for comorbidity. An observed strong significant association between age and total health care costs did not persist when controlling for frailty. Among the five symptoms of frailty, the presence of low activity, exhaustion, and weight loss were associated with higher total costs, with unintentional weight loss and exhaustion having the greatest effects even after controlling for comorbidity.

Except for Hastings et al., who found no association between frailty and health care utilization in the outpatient sector [15], most previous studies generally suggested a positive association between frailty and health care utilization in all health care sectors [9, 16–19]. However, as these studies mainly focused on ‘health care utilization’, the relationship between frailty and corresponding costs has rarely been examined. Thus, our study is in line with the literature. It contributes to the literature by providing further evidence on the association between frailty and costs for a population-based larger sample. One recent study [20] also investigated frailty and comprehensive health care costs, but analyses were limited to a rather small sample of 115 cognitively impaired older adults. For this specific group, frailty was an important driver in particular of informal care costs, whereas the authors did not find significant differences for frailty statuses in formal health care costs.

Beyond that, Peters et al. [21] evaluated the predictive validity of a frailty measure on health care costs. They showed that frailty was a significant predictor of total costs, and in particular long-term care costs. However, frailty was not found to be a predictor of costs of “curative care”, which included inpatient and outpatient physician care and pharmaceuticals. In contrast to our cross-sectional study, participants in Peters et al. were older (on average 80 years vs. 70 years), and much more often institutionalized (36 % vs. less than 1 %). In our study, frailty and nursing care costs were not significantly associated after controlling for comorbidity probably due to the small proportion of nursing care costs with high variance (on average €42 ± €471 for a 3-month period). Besides, measure of frailty in Peters et al. (Groningen Frailty Indicator, GFI) differed from our study, with the GFI being self-assessed and already including aspects of comorbidity and psychosocial as well as cognitive dimensions.

Beyond the costing studies cited above, our study adds insights into the relationship of single frailty symptoms and health care costs. Table 5 shows that each frailty symptom contributes to sectoral and total costs differently. While the relationship among the frailty symptoms has been examined [56], their association with costs was unclear. Our results underline that the term ‘frailty’ covers a heterogeneous group with each symptom having a specific relation towards sectoral and total costs.

A limiting factor of our study was that data on health service utilization were self-reported by participants; therefore recall bias cannot not be ruled out. In addition, although not excluded from participation in the ESTHER study, there were only two participants living in nursing homes and three participants living in assisted living facilities, limiting the representativeness of the study and impeding any conclusions for institutionalized persons. Participants were only included when agreeing to pass the geriatric assessments. Thus, effects of sample selection might have influenced cost estimates. This might especially be the case for persons suffering from a high level of multimorbidity or frailty who potentially refrained from participating. Thus, cost estimates of frailty in this study are likely to be rather conservative, which is, for instance, indicated by the low number of participants with a frailty index of 5, or the mean age of about 70 years without considering age brackets older than 84. A particular strength of our study was the use of population-independent cut points so that the prevalence of frailty did not rely on the specific sample, facilitating comparison with other studies.

Participants of our sample were slightly more often married than people of comparable age from the entire German population: 74 % vs. about 66 % [57]. The educational level, based on the same classification in three groups that we used, could not be compared to the entire population data, as it is not available. For the remaining control variables (sex, age and type of health insurance), there were only slight differences between our sample and the entire population for the cohort aged 57 to 84. This indicates broad representativeness of the 8-year follow-up with regard to the considered socio-economic characteristics. We did not include the variable ‘income’ for two reasons: first, the idea of representing individual economic capacity is already partly captured by the educational level and type of health insurance. Second, the collection in surveys is difficult, leading to potential inaccuracy and missing values [58].

## Conclusions

The study provides evidence that frailty is associated with increased health care costs. The analyses furthermore indicate that frailty is an important factor independent from pure age and comorbidity for analyzing health care costs. Costs were rather attributable to frailty (and comorbidity) than to age. This stresses that the overlapping concepts of multimorbidity and frailty are both necessary to explain health care use and corresponding costs among older adults.

This study furthermore underlines the health economic importance of frailty. The phenomenon of frailty will certainly require more attention in the aging societies of industrialized countries and be a major task for their health care systems.

Future research should further analyze the relationship of frailty and costs by using broader definitions of frailty that include e.g. emotional dimensions. Beyond that, future studies based on panel data should examine the longitudinal relationship between frailty, comorbidity and health care costs.

## Availability of data and materials

Data protection standards and assurances made as part of the informed consent procedure of ESTHER preclude the publication of the source data in publicly available repositories. However, individual data access may be granted within a framework of scientific cooperation.

## Additional file

Additional file 1: Questionnaire. (PDF 93 kb)

## References

[1] OECD (2012). Health Data 2012.

[2] Fortin M, Bravo G, Hudon C, Vanasse A, Lapointe L (2005). Prevalence of multimorbidity among adults seen in family practice. Ann. Fam. Med..

[3] Van den Akker M, Buntinx F, Metsemakers JF, Roos S, Knottnerus JA (1998). Multimorbidity in general practice: prevalence, incidence, and determinants of co-occurring chronic and recurrent diseases. J Clin Epidemiol.

[4] Lehnert T, Heider D, Leicht H, Heinrich S, Corrieri S, Luppa M, Riedel-Heller S, König HH. Review: health care utilization and costs of elderly persons with multiple chronic conditions. Med Care Res Rev. 2011;68(4):387–420. 10.1177/1077558711399580 21813576

[5] Collard RM, Boter H, Schoevers RA, Oude Voshaar RC (2012). Prevalence of Frailty in Community‐Dwelling Older Persons: A Systematic Review. J Am Geriatr Soc.

[6] Bergman H, Ferrucci L, Guralnik J, Hogan DB, Hummel S, Karunananthan S, Wolfson C. Frailty: an emerging research and clinical paradigm—issues and controversies. J Gerontol Ser A Biol Sci Med Sci. 2007;62(7):731–7. 10.1093/gerona/62.7.731 PMC2645660 17634320

[7] Rockwood K (2005). What would make a definition of frailty successful?. Age Ageing.

[8] Gobbens RJ, Luijkx KG, Wijnen-Sponselee MT, Schols JM (2010). Toward a conceptual definition of frail community dwelling older people. Nurs Outlook.

[9] Fried LP, Tangen CM, Walston J, Newman AB, Hirsch C, Gottdiener J, Seeman T, Tracy R, Kop WJ, Burke G. Frailty in older adults evidence for a phenotype. J Gerontol Ser A Biol Sci Med Sci. 2001;56(3):M146–57. 10.1093/gerona/56.3.m146 11253156

[10] Hogan DB, MacKnight C, Bergman H (2003). Models, definitions, and criteria of frailty. Aging Clin Exp Res.

[11] Ensrud KE, Ewing SK, Taylor BC, Fink HA, Stone KL, Cauley JA, Tracy JK, Hochberg MC, Rodondi N, Cawthon PM. Frailty and risk of falls, fracture, and mortality in older women: the study of osteoporotic fractures. J Gerontol Ser A Biol Sci Med Sci. 2007;62(7):744–51. 10.1093/gerona/62.7.744 17634322

[12] Mitnitski A, Song X, Skoog I, Broe G, Cox JL, Grunfeld E, Rockwood K. Relative fitness and frailty of elderly men and women in developed countries and their relationship with mortality. J Am Geriatr Soc. 2005;53(12):2184–9. 10.1111/j.1532-5415.2005.00506.x 16398907

[13] Cawthon PM, Marshall LM, Michael Y, Dam TT, Ensrud KE, Barrett‐Connor E, Orwoll ES. Frailty in older men: prevalence, progression, and relationship with mortality. J Am Geriatr Soc. 2007;55(8):1216–23. 10.1111/j.1532-5415.2007.01259.x 17661960

[14] Saum K-U, Dieffenbach AK, Müller H, Holleczek B, Hauer K, Brenner H (2014). Frailty prevalence and 10-year survival in community-dwelling older adults: results from the ESTHER cohort study. Eur J Epidemiol.

[15] Hastings SN, Purser JL, Johnson KS, Sloane RJ, Whitson HE (2008). Frailty predicts some but not all adverse outcomes in older adults discharged from the emergency department. J Am Geriatr Soc.

[16] Gobbens RJ, van Assen MA, Luijkx KG, Schols JM (2012). The predictive validity of the Tilburg Frailty Indicator: Disability, health care utilization, and quality of life in a population at risk. The Gerontologist.

[17] Robinson TN, Wu DS, Stiegmann GV, Moss M (2011). Frailty predicts increased hospital and six-month healthcare cost following colorectal surgery in older adults. Am J Surg.

[18] Gobbens RJ, van Assen MA, Luijkx KG, Schols JM (2012). Testing an integral conceptual model of frailty. J Adv Nurs.

[19] Fried LP, Ferrucci L, Darer J, Williamson JD, Anderson G (2004). Untangling the concepts of disability, frailty, and comorbidity: implications for improved targeting and care. J Gerontol Ser A Biol Sci Med Sci.

[20] Butler A, Gallagher D, Gillespie P, Crosby L, Ryan D, Lacey L, Coen R, O’Shea E, Lawlor B (2015). Frailty: a costly phenomenon in caring for elders with cognitive impairment. Int. J. Geriatr. Psychiatry..

[21] Peters LL, Burgerhof JG, Boter H, Wild B, Buskens E, Slaets JP (2015). Predictive validity of a frailty measure (GFI) and a case complexity measure (IM-E-SA) on healthcare costs in an elderly population. J Psychosom Res.

[22] Löw M, Stegmaier C, Ziegler H, Rothenbacher D, Brenner H (2004). Epidemiological investigations of the chances of preventing, recognizing early and optimally treating chronic diseases in an elderly population (ESTHER study). Dtsch Med Wochenschr.

[23] Saum K-U, Müller H, Stegmaier C, Hauer K, Raum E, Brenner H (2012). Development and Evaluation of a Modification of the Fried Frailty Criteria Using Population-Independent Cutpoints. J Am Geriatr Soc.

[24] Schöttker B, Saum K-U, Perna L, Ordóñez-Mena JM, Holleczek B, Brenner H (2014). Is vitamin D deficiency a cause of increased morbidity and mortality at older age or simply an indicator of poor health?. Eur J Epidemiol.

[25] König H, Lehnert T, Brenner H, Schöttker B, Quinzler R, Haefeli W, Matschinger H, Heider D. Health service use and costs associated with excess weight in older adults in Germany. *Age Ageing, in press*. 10.1093/ageing/afu120 25829392

[26] Drummond MF, Sculpher MJ, Torrance GW (2005). Methods for the Economic Evaluation of Health Care Programs.

[27] Luce BR, Manning WG, Siegel JE, Lipscomb J, Gold M, Siegel J, Russell L, Weinstein M (1996). Estimating Costs in Cost-Effectiveness Analysis. Cost-Effectiveness in Health and Medicine.

[28] König HH, Born A, Heider D, Matschinger H, Heinrich S, Riedel-Heller SG, Surall D, Angermeyer MC, Roick C. Cost-effectiveness of a primary care model for anxiety disorders. Br J Psychiatry. 2009;195(4):308–17. 10.1192/bjp.bp.108.058032 19794198

[29] Roick C, Kilian R, Matschinger H, Bernert S, Mory C, Angermeyer MC (2001). German adaptation of the client sociodemographic and service receipt inventory - an instrument for the cost of mental health care. Psychiatr Prax.

[30] Folstein MF, Folstein SE, McHugh PR (1975). “Mini-mental state”: a practical method for grading the cognitive state of patients for the clinician. J Psychiatr Res.

[31] Deutsche Krankenhausgesellschaft (2009). Bestandsaufnahme zur Krankenhausplanung und Investitionsfinanzierung in den Bundesländern.

[32] Federal Statistical Office (2008). Grunddaten der Krankenhäuser.

[33] Federal Statistical Office (2008). Kostennachweis der Krankenhäuser.

[34] Krauth C, Hessel F, Hansmeier T, Wasem J, Seitz R, Schweikert B (2005). Empirische Bewertungsansätze in der gesundheitsökonomischen Evaluation - ein Vorschlag der AG Methoden der gesundheitsökonomischen Evaluation (AG MEG). Gesundheitswesen.

[35] Verband der Ersatzkassen (2001). Vergütungslisten für logopädische/sprachtherapeutische Leistungen.

[36] Verband der Ersatzkassen (2002). Vergütungsliste für ergotherapeutische Leistungen.

[37] Verband der Ersatzkassen (2007). Vergütungsliste für podologische Leistungen.

[38] Bundesverwaltungsamt (2011). Gebührenverzeichnis Für Heilpraktiker (GebüH) Und Beihilfefähige Höchstbeträge.

[39] Festbeträge [https://www.gkv-spitzenverband.de/krankenversicherung/hilfsmittel/festbetraege_3/festbetraege.jsp].

[40] Kassenzahnärztliche Bundesvereinigung (2009). Abrechnungshilfe für Festzuschüsse.

[41] Medizinverlag Medizinische Medien Informations GmbH (2011). MMI-Pharmindex 2009.

[42] Federal Statistical Office (2008). Pflegestatistik 2007.

[43] 2008: Verdienste in Deutschland und Arbeitskosten im EU-Vergleich. Press release no. 179 [https://www.destatis.de/DE/PresseService/Presse/Pressekonferenzen/2009/Verdienste/pm_verdienste_PDF.pdf?__blob=publicationFile].

[44] Federal Statistical Office (2010). Verdienste und Arbeitskosten.

[45] Heider D, Matschinger H, Müller H, Saum K-U, Quinzler R, Haefeli WE, Wild B, Lehnert T, Brenner H, König H-H. Health care costs in the elderly in Germany: an analysis applying Andersen’s behavioral model of health care utilization. BMC Health Serv Res. 2014;14(1):71. 10.1186/1472-6963-14-71 PMC3927831 24524754

[46] Guralnik JM, Simonsick EM, Ferrucci L, Glynn RJ, Berkman LF, Blazer DG, Scherr PA, Wallace RB. A short physical performance battery assessing lower extremity function: association with self-reported disability and prediction of mortality and nursing home admission. J Gerontol. 1994;49:M85–94. 10.1093/geronj/49.2.m85 8126356

[47] Radloff LS (1977). The CES-D scale a self-report depression scale for research in the general population. Appl Psychol Meas.

[48] Voorrips LE, Ravelli AC, Petra C, Dongelmans A, Deurenberg P, van Staveren WA (1991). A physical activity questionnaire for the elderly. Med Sci Sports Exerc.

[49] Linn BS, Linn MW, Gurel L (1968). Cumulative illness rating scale. J Am Geriatr Soc.

[50] Miller M, Paradis C, Houck P, Mazumdar S, Stack J, Rifai A, Mulsant B, Reynolds C. Rating chronic medical illness burden in geropsychiatric practice and research: application of the Cumulative Illness Rating Scale. Psychiatry Res. 1992;41(3):237–48. 10.1016/0165-1781(92)90005-n 1594710

[51] Manning WG, Basu A, Mullahy J (2005). Generalized modeling approaches to risk adjustment of skewed outcomes data. J Health Econ.

[52] Diehr P, Yanez D, Ash A, Hornbrook M, Lin D (1999). Methods for analyzing health care utilization and costs. Annu Rev Public Health.

[53] O’Donnell OA, Wagstaff A (2008). Analyzing health equity using household survey data: a guide to techniques and their implementation.

[54] Belotti F, Deb P, Manning WG, Norton EC (2015). twopm: Two-part models. Stata J.

[55] Wooldridge JM (2010). Econometric analysis of cross section and panel data.

[56] Sourial N, Wolfson C, Bergman H, Zhu B, Karunananthan S, Quail J, Fletcher J, Weiss D, Bandeen-Roche K, Béland F. A correspondence analysis revealed frailty deficits aggregate and are multidimensional. J Clin Epidemiol. 2010;63(6):647–54. 10.1016/j.jclinepi.2009.08.007 PMC3718711 19880286

[57] Federal Statistical Office. Statistisches Jahrbuch 2012. Wiesbaden: Statistisches Bundesamt; 2012.

[58] Moore JC, Stinson LL, Welniak EJ (2000). Income measurement error in surveys: A review. J. Off. Stat. Stockh.

