# Supplementary material for: Associations of frailty with health care costs – results of the ESTHER cohort study
Source: BMC Health Serv Res. 2016 Apr 14;16:128. doi: 10.1186/s12913-016-1360-3 (PMC4831082; doi:10.1186/s12913-016-1360-3)
Supplement: Additional file 1: — Questionnaire. (PDF 93 kb) [file 12913_2016_1360_MOESM1_ESM.pdf]

Gesundheitsökonomie

0. Angaben zur Kranken- und Pflegeversicherung

Wie sind Sie krankenversichert? *Bitte machen Sie nur ein Kreuz.*

☐ Gesetzliche Krankenversicherung ☐ Keine Krankenversicherung
☐ Private Krankenvollversicherung einschließlich Beihilfe ☐ Weiß nicht
☐ Sonstige Krankenversicherung

Haben Sie eine private Kranken<u>zusatz</u>versicherung?
Wenn ja, wie hoch sind die monatlichen Prämien (Beiträge)

☐ NEIN ☐ JA ☐ Weiß nicht |__|__|__|__| Euro

Wie sind Sie pflegeversichert? *Bitte machen Sie nur ein Kreuz.*

☐ Gesetzliche Pflegeversicherung ☐ Private Pflegepflichtversicherung
☐ Keine Pflegeversicherung ☐ Weiß nicht

Haben Sie eine private Pflegezusatzversicherung?
Wenn ja, wie hoch sind die monatlichen Prämien (Beiträge)

☐ NEIN ☐ JA ☐ Weiß nicht |__|__|__|__| Euro

Falls gesetzlich krankenversichert, sind Sie von Zuzahlungen (z.B. für Medikamente etc.) befreit?

☐ NEIN ☐ JA

1. Stationäre Versorgung

Waren Sie in den letzten **3 Monaten** zur stationären oder **teil**stationären (Tagesklinik) Behandlung in einem Allgemeinkrankenhaus, einem psychiatrischen Krankenhaus oder einer Rehabilitationseinrichtung?

☐ NEIN ☐ JA

Wenn ja, tragen Sie bitte jeweils die Anzahl der **Tage** ein, die Sie dort in den letzten **3 Monaten** waren. (Falls Sie die ganzen 3 Monate in einer Einrichtung waren, tragen Sie bitte „90“ ein. Tragen Sie bitte „0“ ein, wenn Sie in einer Einrichtung nicht in Behandlung waren. Zählen Sie bei teilstationären Aufenthalten bitte Wochenenden und behandlungsfreie Tage nicht mit)

Allgemeinkrankenhaus	Psychiatrisches Krankenhaus	Kur- oder Rehabilitationsklinik
|__|__| Tage ☐ unbekannt	|__|__| Tage ☐ unbekannt	|__|__| Tage ☐ unbekannt

Wie viel haben sie in den letzten 3 Monaten insgesamt für die von Ihnen genannten stationären und teilstationären Versorgungsleistungen aus eigener Tasche gezahlt oder zugezahlt?

|__|__|__|__| Euro ☐ unbekannt

2. Ambulante ärztliche und psychologische Versorgung

Bitte geben Sie an, ob und wie oft Sie die folgenden Ärzte in den letzten 3 Monaten besucht haben. Geben Sie bitte auch Hausbesuche an.

				unbekannt					unb.
Allgemeinmedizi-ner bzw. Hausarzt	☐ NEIN	☐ JA	|__|__|	☐	Gynäkologe	☐ NEIN	☐ JA	|__|__|	☐
Kardiologe	☐ NEIN	☐ JA	|__|__|	☐	Urologe	☐ NEIN	☐ JA	|__|__|	☐
Psychiater	☐ NEIN	☐ JA	|__|__|	☐	Neurologe	☐ NEIN	☐ JA	|__|__|	☐
Orthopäde	☐ NEIN	☐ JA	|__|__|	☐	Zahnarzt	☐ NEIN	☐ JA	|__|__|	☐
HNO-Arzt	☐ NEIN	☐ JA	|__|__|	☐	Radiologe	☐ NEIN	☐ JA	|__|__|	☐
Hautarzt	☐ NEIN	☐ JA	|__|__|	☐	Psychologe/ Psychotherapeut	☐ NEIN	☐ JA	|__|__|	☐
Augenarzt	☐ NEIN	☐ JA	|__|__|	☐	Notarzt	☐ NEIN	☐ JA	|__|__|	☐
					Sonstige Ärzte	☐ NEIN	☐ JA	|__|__|	☐

3. Ambulante nichtärztliche Versorgung

Bitte geben Sie an, ob und wie oft Sie die folgenden Therapeuten in den letzten 3 Monaten besucht haben. Geben Sie bitte auch Hausbesuche an.

				unbekannt					unb.
Heilpraktiker	☐ NEIN	☐ JA	|__|__|	☐	Physiotherapeut (Krankengym-nastik, Ergothe-rapie, Massage etc.)	☐ NEIN	☐ JA	|__|__|	☐

Wenn ja, wie viel haben Sie in den letzten 3 Monaten insgesamt für die von Ihnen genannten ambulanten Versorgungsleistungen aus eigener Tasche gezahlt oder zugezahlt?

|__|__|__|__| Euro ☐ unbekannt

+

4. Pflegeheim, teilstationäre Pflege, betreutes Wohnen, Altenheim

Haben Sie in den letzten 3 Monaten einen Aufenthalt in einem Pflegeheim, in einer Einrichtung der teilstationären Pflege, dem betreuten Wohnen oder einem Altenheim gehabt?

☐ NEIN ☐ JA

Wenn ja, tragen Sie bitte jeweils die Anzahl der **Tage** ein, die Sie dort in den letzten **3 Monaten** untergebracht waren. (Falls Sie die ganzen 3 Monate dort gelebt haben, tragen Sie bitte „90“ ein. Tragen Sie bitte „0“ ein, wenn Sie in einer Einrichtung keinen Aufenthalt hatten.)

Pflegeheim	Teilstationäre Pflege	Altenheim	betreutes Wohnen
|__|__| Tage ☐ unb.	|__|__| Tage ☐ unb.	|__|__| Tage ☐ unb.	|__|__| Tage ☐ unb.

+

5. Ambulante Pflege und Hilfen im Alltag durch professionelle Dienste

Wurden Sie in den letzten 3 Monaten krankheits- oder altersbedingt durch ambulante professionelle Dienste gepflegt bzw. mussten Sie deren Hilfe krankheits- oder altersbedingt für Tätigkeiten wie die Haushaltsführung in Anspruch nehmen?

☐ NEIN ☐ JA

Wenn ja, füllen Sie bitte in der untenstehenden Tabelle für jede in Anspruch genommene professionelle Dienstleistung eine Zeile aus.

	Haben Sie diese Leistung in Anspruch genommen?	An wie vielen **Tagen**? *(Falls die ganzen 3 Monate, tragen Sie bitte „90“ ein)*	Wie viele **Minuten** hat die Dienstleistung durchschnittlich **pro Tag** gedauert?
Ambulanter Pflegedienst (Grund- und Behandlungspflege)	☐ NEIN ☐ JA	|__|__| ☐ unbekannt	|__|__|__|__| ☐ unb.
Haushaltshilfe	☐ NEIN ☐ JA	|__|__| ☐ unbekannt	|__|__|__|__| ☐ unb.
Essen auf Rädern	☐ NEIN ☐ JA	|__|__| ☐ unbekannt	

+

+

**6. Pflege und Hilfen im Alltag durch Familienangehörige, Freunde, Bekannte oder Nachbarn**<br>Wurden Sie in den letzten 3 Monaten krankheits- oder altersbedingt durch Familienangehörige, Freunde, Bekannte oder Nachbarn gepflegt bzw. mussten sie deren Hilfe krankheits- oder altersbedingt für Tätigkeiten wie die Haushaltsführung in Anspruch nehmen?<br>☐ JA ☐ NEIN
Wenn ja, an wie vielen Tagen wurden Sie in den letzten 3 Monaten krankheits- oder altersbedingt von Familienangehörigen, Freunden, Bekannten oder Nachbarn gepflegt bzw. mussten sie deren Hilfe in Anspruch nehmen? *(Tragen Sie bitte „90“ ein, falls dies für die ganzen 3 Monate zutrifft)*<br>|__|__| Tage ☐ unbekannt
Wie viele Minuten haben die Pflege- und Hilfeleistungen durch Familienangehörige, Freunde, Bekannte oder Nachbarn pro Tag durchschnittlich gedauert?<br>|__|__|__|__| Minuten ☐ unbekannt
Wie viel haben Sie in den letzten 3 Monaten insgesamt für die von Ihnen genannten Pflegeleistungen (Pflegeheim, teilstationäre Pflege, betreutes Wohnen oder Altenheim, professionelle Dienste, Familienangehörige, Freunde, Bekannte oder Nachbarn) aus eigener Tasche gezahlt oder zugezahlt?<br>|__|__|__|__| Euro ☐ unbekannt

+

+

7. Medizinische Hilfsmittel

Bitte geben Sie die medizinischen Hilfsmittel an, die Sie in den letzten 3 Monaten gekauft oder erhalten haben. Bitte geben Sie keine Hilfsmittel an, die Sie ausschließlich im Rahmen eines stationären Aufenthaltes genutzt haben.

	Haben Sie in den letzten 3 Monaten eines dieser Hilfsmittel gekauft oder erhalten?		Wie viel Stück oder Packungen dieser Hilfsmittel haben Sie gekauft oder erhalten?	
Hörgerät	☐ NEIN	☐ JA	|__|__| Stück	☐ unbekannt
Rollstuhl	☐ NEIN	☐ JA	|__|__| Stück	☐ unbekannt
Gehhilfe	☐ NEIN	☐ JA	|__|__| Stück	☐ unbekannt
Brille	☐ NEIN	☐ JA	|__|__| Stück	☐ unbekannt
Verbandsmaterial	☐ NEIN	☐ JA	|__|__| Stück	☐ unbekannt.
Inkontinenzeinlagen	☐ NEIN	☐ JA	|__|__| Stück	☐ unbekannt
Stütz- und Kompressionsstrümpfe	☐ NEIN	☐ JA	|__|__| Stück	☐ unbekannt
medizinische Geräte	☐ NEIN	☐ JA	|__|__| Stück	☐ unbekannt
Blutzuckerteststreifen	☐ NEIN	☐ JA	|__|__| Stück	☐ unbekannt
Sonstige med. Hilfsmittel:	☐ NEIN	☐ JA	|__|__| Stück	☐ unbekannt

Wie viel haben Sie für diese und gegebenenfalls andere Hilfsmittel in den letzten 3 Monaten aus eigener Tasche insgesamt gezahlt oder zugezahlt?

|__|__|__|__|__| Euro ☐ unbekannt

8. Zahnersatz

Haben Sie in den letzten 3 Monaten Zahnersatz gekauft oder erhalten?

☐ JA ☐ NEIN

Wenn ja, wie viel hat der Zahnersatz, den Sie in den letzten 3 Monaten gekauft oder erhalten haben, insgesamt gekostet?

|__|__|__|__|__| Euro ☐ unbekannt

Wie viel haben Sie in den letzten 3 Monaten für Zahnersatz aus eigener Tasche gezahlt oder zugezahlt?

|__|__|__|__|__| Euro ☐ unbekannt

9. Medikamente

Haben Sie in den letzten 3 Monaten regelmäßig oder bei Bedarf Medikamente eingenommen?
(Bitte berücksichtigen Sie Medikamente, die Sie im Rahmen einer vollstationären oder teilstationären Krankenhausbehandlung bzw. einer ebensolchen Kur/Rehabilitationsmaßnahme erhalten haben, **nicht**)

☐ JA ☐ NEIN

Wenn ja, wie viel haben Sie in den letzten 3 Monaten insgesamt für die von Ihnen eingenommenen Medikamente aus eigener Tasche gezahlt oder zugezahlt?

|__|__|__|__| Euro ☐ unbekannt

10. Soziales Netzwerk – quantitative Aspekte

**In den folgenden 3 Fragen geht es um Ihre Familienangehörigen (einschließlich Ehepartner, angeheiratete Verwandte und sonstige Verwandte).**	9 oder mehr	5 bis 8	3 oder 4	2	1	keine
Mit wie vielen Familienangehörigen treffen Sie sich mindestens einmal im Monat oder haben sie mindestens einmal im Monat sonstigen Kontakt (z.B. telefonisch)?	☐	☐	☐	☐	☐	☐
Mit wie vielen Familienangehörigen sind Sie so vertraut, dass Sie sie um Unterstützung bitten können?	☐	☐	☐	☐	☐	☐
Mit wie vielen Familienangehörigen sind Sie so vertraut, dass Sie private Angelegenheiten mit ihnen besprechen können?	☐	☐	☐	☐	☐	☐
**In den folgenden 3 Fragen geht es um Ihre Kontakte zu Freunden und Nachbarn.**						
Mit wie vielen Freunden oder Nachbarn treffen Sie sich mindestens einmal im Monat oder haben sie mindestens einmal im Monat sonstigen Kontakt (z.B. telefonisch)?	☐	☐	☐	☐	☐	☐
Mit wie vielen Freunden oder Nachbarn sind Sie so vertraut, dass Sie sie um Unterstützung bitten können?	☐	☐	☐	☐	☐	☐
Mit wie vielen Freunden oder Nachbarn sind Sie so vertraut, dass Sie private Angelegenheiten mit ihnen besprechen können?	☐	☐	☐	☐	☐	☐

11. Soziales Netzwerk – qualitative Aspekte

		fast nie	gelegentlich	oft	täglich	mehrmals täglich
1.	Wie häufig unterstützt Sie jemand im Alltag?	☐	☐	☐	☐	☐
2.	Wie häufig unterstützt Sie jemand bei schwierigen Entscheidungen und Sorgen?	☐	☐	☐	☐	☐
3.	Wenn Sie ein Gespräch brauchen, wie häufig gibt es jemand, der Ihnen richtig zuhört?	☐	☐	☐	☐	☐
4.	Wie häufig zeigt Ihnen jemand seine Liebe oder Zuneigung?	☐	☐	☐	☐	☐
5.	Wie häufig unterstützen Sie selbst andere Menschen mit Rat und Tat?	☐	☐	☐	☐	☐

+

12. Maximale Zahlungsbereitschaft für Krankenversicherung

Stellen Sie sich vor, Sie wären nicht krankenversichert:

Unter Berücksichtigung Ihres Netto-Haushaltseinkommens, wie viel Euro wären Sie maximal bereit, monatlich für eine Krankenversicherung zu zahlen, wenn diese den gleichen Leistungsumfang wie Ihre derzeitige Krankenversicherung hätte?

Bitte betrachten Sie die untenstehende Tabelle.

1. In Spalte A, kreuzen Sie bitte alle Werte an, die Sie auf jeden Fall für eine Krankenversicherung zahlen würden. Beginnen Sie mit dem niedrigsten Wert und hören Sie mit dem höchsten Wert auf, den Sie zu zahlen bereit wären.
2. In Spalte B, kreuzen bitte Sie alle Werte an, die Sie auf keinen Fall für eine Krankenversicherung zahlen würden. Beginnen Sie mit dem höchsten Wert und hören Sie mit dem niedrigsten Wert auf, den Sie nicht zahlen würden.
3. Bitte überspringen Sie diejenigen Werte, bei denen Sie sich unsicher sind. Dies gilt sowohl für Spalte A als auch für Spalte B.

+

	**Spalte A**<br>Ich würde monatlich definitiv zahlen (↓)	**Spalte B**<br>Ich würde monatlich definitiv **nicht** zahlen (↑)
50 Euro	☐	☐
100 Euro	☐	☐
200 Euro	☐	☐
300 Euro	☐	☐
400 Euro	☐	☐
500 Euro	☐	☐
750 Euro	☐	☐
1000 Euro	☐	☐
Mehr als 1000 Euro	☐	☐

Proxy anwesend: ☐ Ja ☐ Nein

Gesundheitsökonomie – Fragen beantwortet:
- ☐ Studienteilnehmer allein
- ☐ mit Proxy gemeinsam
- ☐ Proxy allein

+
